# A Hydrophobic Small Protein, BpOF4_01690, Is Critical for Alkaliphily of Alkaliphilic *Bacillus pseudofirmus* OF4

**DOI:** 10.3389/fmicb.2018.01994

**Published:** 2018-08-28

**Authors:** Tetsuaki Takahashi, Terry A. Krulwich, Masahiro Ito

**Affiliations:** ^1^Graduate School of Life Sciences, Toyo University, Gunma, Japan; ^2^Department of Pharmacological Sciences, Icahn School of Medicine at Mount Sinai, New York, NY, United States; ^3^Bio-Nano Electronics Research Centre, Toyo University, Kawagoe, Japan

**Keywords:** alkaliphiles, small protein, *Bacillus pseudofirmus*, respiratory chain, pH homeostasis, alkaliphily

## Abstract

A monocistronic small protein, BpOF4_01690, was annotated in alkaliphilic *Bacillus pseudofirmus* OF4. It comprises 59 amino acids and is hydrophobic. Importantly, homologs of this protein were identified only in alkaliphiles. In this study, a mutant with a BpOF4_01690 gene deletion (designated Δ01690) exhibited weaker growth than that of the wild type in both malate-based defined and glucose-based defined media under low-sodium conditions at pH 10.5. Additionally, the enzymatic activity of the respiratory chain of Δ01690 was much lower than that of the wild type. These phenotypes were similar to those of a *ctaD* deletion mutant and an *atpB-F* deletion mutant. Therefore, we hypothesize that BpOF4_01690 plays a critical role in oxidative phosphorylation under highly alkaline conditions.

## Introduction

**FIGURE 1:**
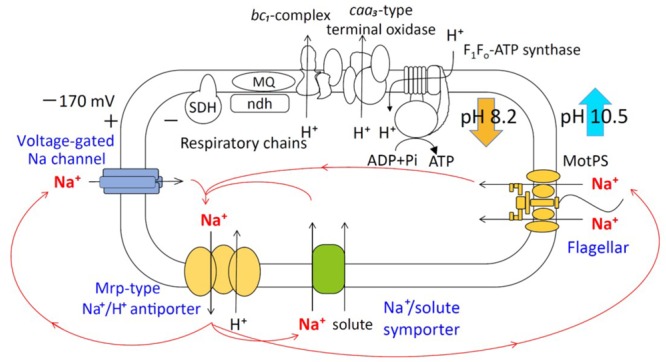
Schematic diagram of the Na^+^ cycle of alkaliphilic *Bacillus pseudofirmus* OF4. Alkaliphilic *B. pseudofirmus* OF4 grows well around pH 10, and the intracellular pH is maintained around 8.2. The Mrp-type Na^+^/H^+^ antiporter performs a crucial role in the maintenance of intracellular ion concentrations and pH homeostasis. Na^+^/H^+^ antiporters catalyze proton accumulation in the cytoplasm while cells are extruding H^+^ during respiration. Na^+^ re-entry in support of pH homeostasis is achieved by Na^+^: solute symporters. When Na^+^ entry is limited, e.g., at low [Na^+^] concentrations or a lack of symporter substrates, the voltage-gated Na channel operates as a physiologically important ensuring a re-entry route for Na^+^. The Na^+^-driven flagellar motor (MotPS channel) functions as a secondary pathway. SDH, succinate dehydrogenase; MQ, menaquinone; Ndh-II, type II NADH:quinone oxidoreductase.

Alkaliphilic microorganisms usually grow vigorously in highly alkaline environments and require Na^+^ for their growth (Horikoshi, 1991; Krulwich et al., 2011; Preiss et al., 2015). Na^+^ cycling was found to be critical for the alkaline pH adaptation of alkaliphilic bacteria (Ito et al., 2004a,b) (**Figure 1**). Although it is extremely difficult to produce and utilize a proton-motive force (PMF) at highly alkaline pH, ATP synthesis by oxidative phosphorylation (OXPHOS) using F_1_F_o_-ATP synthase is driven by PMF in alkaliphilic *Bacillus* species (Guffanti and Krulwich, 1994). Therefore, some effective ATP synthesis mechanisms are expected to operate in these bacteria in their highly alkaline environment. It has been suggested that accumulation of protons on the outer surface of the cytoplasmic membrane (Yoshimune et al., 2010) facilitates energy coupling that is more efficient than usual, thereby increasing the feasibility of ATP synthesis in a highly alkaline pH environment (Krulwich, 1995). The alkaliphilic *Bacillus clarkia* K24-1U was also proposed to efflux protons by the respiratory chain, accumulating them on the outer surface of the cytoplasmic membrane (Cherepanov et al., 2003; Mulkidjanian, 2006). Another possibility is the activity of an unidentified proton carrier that depends on the dielectric properties of the membrane potential (Liu et al., 2007). Thus, the outer surface vicinity of the cytoplasmic membrane is locally acidified, and enough PMF necessary for the synthesis of ATP is provided despite the alkaline environment. Fast cardiolipin-mediated proton translocation from the respiratory chain pumps to ATP synthase by OXPHOS was also hypothesized. However, the mutational loss of membrane cardiolipin did not significantly affect alkaliphile ATP synthesis in alkaliphilic *B. pseudofirmus* OF4 (Liu et al., 2014).

Descriptions of a unique “alkaliphily” motif in the *c*-ring of ATP synthase from alkaliphilic *B. pseudofirmus* OF4 had been noted in earlier studies of alkaliphilic bacteria (Liu et al., 2009; Fujisawa et al., 2010). This alkaliphile OXPHOS motif could underpin the efflux of protons by the respiratory chain. Nonetheless, the amount of protons is not in equilibrium with that of the external environment. Consequently, during ATP synthesis, protons are directly transferred to F_1_F_o_-ATP synthase through the cytoplasmic membrane. Results of differential scanning calorimetry analysis and saturation transfer electron spin resonance provided indirect evidence for the interaction between the *caa*_3_-type terminal oxidase and F_1_F_o_-ATP synthase in the proteoliposome (Liu et al., 2007). However, no reports demonstrate the presence of a direct interaction between *caa*_3_-type oxidase and F_1_F_o_-ATP synthase.

**FIGURE 2:**
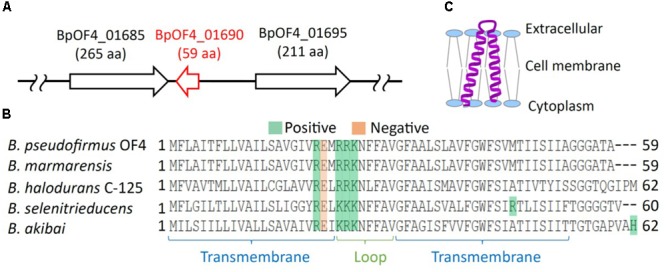
Features of BpOF4_01690. **(A)** The arrangement of BpOF4_01690 and the surrounding genes. **(B)** Multiple alignment analysis of BpOF4_01690 and its homologous proteins was performed using Uniprot (http://www.uniprot.org/). The positively charged amino acid residues are denoted in green, whereas the negatively charged amino acid residues are shown in pink. Transmembrane region prediction was performed using SOSUI (http://harrier.nagahama-i-bio.ac.jp/sosui/) and TMHMM server ver. 2 (http://www.cbs.dtu.dk/services/TMHMM/). **(C)** Estimated schematic diagram of BpOF4_ 01690 from the secondary protein structure prediction and hydropathy profile.

The hypothetical small protein BH2819 containing 62 amino acids was identified as a complementation gene product of an alkaline pH-sensitive mutant, which was isolated from alkaliphilic *B. halodurans* C-125 by chemical mutagenesis using ethyl methanesulfonate (Aono et al., 1993). Since the BH2819 mutants showed both decreased NADH oxidase activity and loss of growth at a highly alkaline pH, the potential involvement of the BH2819 protein in alkali mechanisms attracted scientific interest, particularly regarding the respiratory chain complexes. The previous evidence of a lack of genetic accessibility of the target gene disruption technique of *B. halodurans* C-125 genomic DNA, whole genome sequencing of *B. pseudofirmus* OF4, a closely related species to *B. halodurans* C-125, was performed. The result revealed a homologous protein of BH2819, designated BpOF4_01690, a monocistronic small protein, which was unique and found mostly in alkaliphilic *Bacillus* species (**Figure 2**) (Janto et al., 2011). BpOF4_01690 is a low-molecular-weight protein that, similarly to the BH2819 protein, consists of only 59 amino acids (GenBank accession no. ADC48406.1).

In major studies on small proteins reported by Hobbs et al. (2011) and Storz et al. (2014), this type of protein was defined as proteins made up of <50 amino acids (aa’s). However, we encountered a somewhat larger protein in alkaliphilic *B. pseudofirmus* OF4: BpOF4_01690 with 59 aa’s and a similar protein from *B. halodurans* C-125, BH2819 with 62 aa’s. While not quite as small as the “small proteins” studied by others, they appeared to be sufficiently small to be worthwhile examining in this context.

Many small proteins studied to date are classified as integral membrane proteins. The function of small proteins is diverse, including spore formation, cell division, transport, and the activities of membrane-bound enzymes, such as we were studying, as well as protein kinases, and signal transduction systems (Su et al., 2013; Storz et al., 2014). It has been reported that the small protein Rcf1 plays an important role in the formation of respiratory chain supercomplexes of mitochondria (Chen et al., 2012). Rcf1 is interposed between complex III and IV, and its function is to promote the formation of the supercomplex. Consequently, respiratory failure occurs in the mitochondrial respiratory chains of Rcf1-defective mutants.

In the present study, we used *B. pseudofirmus* OF4, which was successfully subjected to genome engineering. First, a BpOF4_01690-deleted strain (named Δ01690) was constructed from *B. pseudofirmus* OF4. Then, growth experiments at neutral and alkaline pH and several Na^+^ concentrations were conducted. In addition, media with different carbon sources were examined in the wild type *B. pseudofirmus* OF4-811M and in the Δ01690 mutant. The activities of the respiratory chain complexes of the wild type and Δ01690 mutant were also compared. This investigation is aimed at identifying the physiological role of small protein BpOF4_01690 at highly alkaline pH.

## Materials and Methods

### Bacterial Strains and Plasmids

The bacterial strains and plasmids used in the present study are listed in **Table 1**, and the primers utilized in our investigation are available on request. The wild type strain was alkaliphilic *B. pseudofirmus* OF4 (Clejan et al., 1989), whose whole genome had been previously sequenced (Janto et al., 2011). The BpOF4_01690 gene and *ctaD* (accession no. BpOF4_00910) deletion mutant were individually constructed in the native alkaliphile host as described previously (Liu et al., 2013). Briefly, to construct the Δ01690 strain, upstream and downstream flanking regions of the BpOF4_01690 gene of approximately 800 bp were amplified using *B. pseudofirmus* OF4 genomic DNA as the template and subsequently cloned into pGEM7Zf(+) (Promega) and pG^+^host4 (Appligene, Pleasanton, CA, United States) sequentially. The resulting pG^+^host4 construct was transformed into the *B. pseudofirmus* OF4 strain by protoplast transformation (Ito et al., 1997). The Δ01690 strain was constructed after a single crossover step and a double crossover recombination step. The deletion region was verified by DNA sequencing performed by Eurofins Genomics K.K. (Tokyo, Japan). Restoration of a functional BpOF4_01690 gene in the mutant strain Δ01690 was achieved by applying a similar strategy to replace the region that was disrupted in the mutants with the sequence of the wild type. The Δ*ctaD* strain was constructed in a similar way. The deleted *atpBEF* (a, c, and b subunit of the F_o_ part of ATPase, accession no. BpOF4_06880, BpOF4_06875, and BpOF4_06870) of *B. pseudofirmus* OF4 was used for the development of the ΔF_o_ strain (Wang et al., 2004). The β-His strain of *B. pseudofirmus* OF4 containing a six-codon addition encoding 6-His just after the N-terminal methionine of the β-subunit of F_1_ part of ATPase (AtpD, accession no. BpOF4_06850) was used for immune blotting and pull-down assay (Fujisawa et al., 2010).

**Table 1:** Bacterial strains and plasmids used in this study.

Strain and plasmid	Genotype and description	Source and reference
***E. coli* strains**		
DH5αMCR	F-*mcrAΔ1 (mrr-hsd RMS-mcrBC) Φ80dlacZ Δ(lacZYAargF) U169 deoR recA1 endA1 supE44 λthi-1 gyr-496 relA1*	Stratagene
XL1-Blue MRF’	*Δ(mcrA)183 Δ(mcrCB-hsdSMR-mrr)173 endA1 supE44 thi-1 recA1 gyrA96 relA1 lac [F’proAB lacIqZDM15 Tn10 (Tetr)]*	GIBCO/BRL
***Bacillus pseudofirmus* OF4 strains**		
811M	Wild type, Met^-^	Clejan et al., 1989
Δ01690	811M, ΔBpOF4_01690	This study
Δ01690-R	Δ01690, BpOF4_01690 restored at the native location	This study
Δ01690-R-His_6_	Δ01690, BpOF4_01690 with 6xHis-tag at C-terminal side restored at the native location	This study
Δ*ctaD*	811M, Δ*ctaD*	This study
ΔF_o_	811M, Δ*atpBEF*	Wang et al., 2004
β-His	811M, AtpD with 6xHis-tag at N-terminal side	Fujisawa et al., 2010
**Plasmids**		
pGEM7Zf(+)	Cloning vector Amp^R^	Promega
pGEM7zf(+)_Δ01690	pGEM7zf(+) plus BpOF4_Δ01690 fragment	This study
pMW118	Cloning vector Amp^R^	Nippon Gene
pMW118_01690-R	pMW118+BpOF4_01690 fragment	This study
pG^+^host4	Temperature-sensitive vector, Erm^R^	Appligene
pG^+^host4_Δ01690	pG^+^host4 + BpOF4_Δ01690 fragment	This study
pG^+^host4_01690-R	pG^+^host4 + BpOF4_01690 fragment	This study
pG^+^host4_01690-R-His_6_	pG^+^host4 + BpOF4_01690 with 6xHis-tag at C-terminal side fragment	This study
pG^+^host4_ΔctaD	pG^+^host4 + Δ*ctaD* fragment	This study

### Growth Media and Conditions

Two types of media were used for the experiments; they were buffered at pH 7.5 and 10.5. Either malate (to 50 mM) was used as the carbon source to support non-fermentative growth or glucose (to 50 mM) to promote fermentative growth. The semi-defined media with the above-mentioned respective carbon sources were referred to as KMYE (potassium malate-yeast extract) and KGYE (potassium glucose-yeast extract) media (Wang et al., 2004). The KMYE medium (pH 10.5) contained 6.70 g of malic acid, 1 g of Yeast Extract, 12.44 g of K_2_CO_3_, 1 g of KHCO_3_, 0.136 g of K_2_HPO_4_, 0.025 g of MgSO_4_⋅7H_2_O, and 1% (v/v) trace elements per liter of deionized water. The pH was adjusted to 10.5 with potassium hydroxide solution. The KMYE medium (pH 7.5) contained 6.70 g of malic acid, 1 g of Yeast Extract, 16.37 g of K_2_HPO_4_, 0.8 g of KH_2_PO_4_, 0.025 g of MgSO_4_⋅7H_2_O, and 1% (v/v) trace elements per liter of deionized water. The pH was adjusted to 7.5 with potassium hydroxide solution. KGYE medium contained the same composition as that of the KMYE medium except for the carbon source which was 9 g of glucose. MYE medium (pH 10.5) was used for growth of the β-His strain. The MYE medium contained 6.70 g of malic acid, 1 g of yeast extract, 9.54 g of Na_2_CO_3_, 0.84 g of NaHCO_3_, 0.136 g of K_2_HPO_4_, 0.025 g of MgSO_4_⋅7H_2_O, and 1% (v/v) trace elements per liter of deionized water (pH 10.5). The pH was adjusted to 10.5 with sodium hydroxide solution. An *E. coli* strain was grown at 30°C for derivatives of pG^+^host4 or 37°C for another plasmid in LB medium. When antibiotics are required for growth selection, the particular medium was supplemented with erythromycin (0.3–0.6 μg/ml for *B. pseudofirmus* and 150–300 μg/ml for *E. coli*) or ampicillin (100 μg/ml). The cells were grown at 37°C with shaking. Their growth was monitored by measuring the absorbance at 600 nm using a spectrophotometer.

### Alignment of the Small Protein With Homologous Proteins of Several Bacterial Species

The amino acid sequences of BpOF4_01690 and several homologs were obtained using the BLASTP algorithm at NCBI^1^. Selected amino acid residues in the alignment were analyzed using ClustalW^2^.

### Isolation of Everted Membrane Vesicles and ATPase Assays

Everted membrane vesicles were prepared from overnight cultures grown under several conditions as described previously (Liu et al., 2014). Protein content was determined by the Lowry method using lysozyme as the standard (Lowry et al., 1951). Octylglucoside-stimulated ATPase assays were performed for 3 min at 37°C and pH 8.0 in a 0.5-ml volume, containing 20 mM Tricine-NaOH, 5 mM ATP (sodium salt, Sigma), 2.5 mM MgCl_2_, 30 mM octylglucoside, 50 mM Na_2_SO_3_, and 20 μg membrane protein (Liu et al., 2014). Subsequently, a 0.5-ml volume of Pi detection solution containing 0.3 ml of LeBel reagent was added, which comprised 1% sodium sulfate, 0.4% 4-(methylamino) phenol, and 1% ammonium molybdate. The reactions were incubated for 5 min at room temperature and terminated by a 0.1-ml volume of 34% sodium citrate. The precipitated protein was removed by centrifugation, and the liberated P_i_ in the supernatants was measured at 750 nm according to Lebel et al. (1978).

### Assays of Respiratory Chain Components

All enzyme assays were performed at room temperature using a Shimadzu UV-1800 UV-Visible spectrophotometer. Tris-HCl (50 mM, pH 8) was utilized as the assay buffer, and 1 ml of 50 or 100 μg of everted membrane vesicle protein was used as the assay volume. NADH oxidase assays were performed by monitoring the decrease of *A*_340_ over time in the presence of 0.2 mM NADH. The NADH-ferricyanide oxidoreductase activity was measured at 420 nm in a buffer containing 1 mM NADH, 1 mM K_3_Fe(CN)_6_, and 10 mM KCN, as described previously (Swartz et al., 2007). Succinate dehydrogenase activity was monitored by following the phenazine methosulfate-coupled reduction of 2,6-dichloroindophenol at 600 nm (Hatefi, 1978). The reaction mixture, consisting of 10 mM succinate, 50 μg vesicles, and 10 mM KCN, was preincubated for 5 min at room temperature. Then, 0.07 mM 2,6-dichloroindophenol and 1.625 mM phenazine methosulfate were added to initiate the reaction. The *N,N,N*’,*N*’-tetramethyl-*p*-phenylenediamine (TMPD) oxidase level was determined by monitoring the increase in *A*_562_ in the presence of 0.25 mM TMPD (Sakamoto et al., 1996). The extinction coefficients (mM^-1^ cm^-1^) used for activity calculations were as follows: 6.2 at 340 nm, 1 at 420 nm, 21 at 600 nm, and 10.5 at 562 nm. One unit (U) was defined as 1 μmol of substrate reduced or oxidized per minute per mg of protein.

### Immunoblot Analysis of BpOF4_01690-6xHis Protein in Strain Δ01690-R-His_6_ Membrane Fractions

Five microliters of membrane suspension (4 μg of membrane protein/μl) from each sample was used for one-dimensional sodium dodecyl sulfate (SDS)-PAGE analyses of the membrane samples. The same volume of SDS loading buffer was added to each sample, after which the proteins were separated on 12% polyacrylamide SDS gels. Next, the gels were electrophoretically transferred to nitrocellulose filters (Bio-Rad) by the application of 60 V for 3 h in Tris-glycine-methanol buffer [25 mM Tris, 192 mM glycine, and 20% (v/v) methanol (pH 8.3)]. The BpOF4_01690-His_6_ protein was detected by anti-His antibody HRP conjugate (Qiagen). ECL solution (Promega) was the usual detection reagent. A quantitative imaging system, Pluor-S MAX (Bio-Rad), was used for the detection and analysis of chemiluminescence images.

### Heme Staining Analysis of Cytochrome Content

For heme staining and subsequent analyses, 30 μg of everted membrane vesicle protein was separated by native 12% PAGE (Schagger and Von Jagow, 1987). The gels were immersed for 30 min at room temperature in the dark in 25 ml of staining solution (pH 4.7) containing 0.5 mg/ml 3,3′,5,5′-tetramethylbenzidine, 50% methanol, and 1 M sodium acetate (Guikema and Sherman, 1981), with slow shaking, after which H_2_O_2_ was added to 0.5%. The stained bands appeared in 5 min, after which the gels were scanned. The bands were quantified by ImageJ 1.47 software and described as % of WT, with WT set at 100%.

### Solubilization of Membrane Proteins from the β-His Strain

Membrane vesicles were prepared from overnight cultures grown in MYE medium at pH 10.5 as described previously (Liu et al., 2009). 10 mg/mL membrane proteins from the β-His strain were solubilized by an extraction solution which contains 200 mM NaCl, 1%(w/v) dodecyl maltoside (DDM), 3 mM HEPES, 15 mM MgCl_2_ and 3% glycerol (pH 8.0). The pH was adjusted to 8.0 with sodium hydroxide solution. The solution was gently mixed with a nutator at 4°C for 1 h. Ultracentrifugation (Beckman Coulter Optima TL 100) was performed at 45,000 rpm at 4°C for 1 h to remove insoluble proteins.

### Sucrose Density Gradient Ultracentrifugation

The Ultra-clear^TM^ centrifuge tube was first filled with 4 ml of 20% sucrose and then 4 ml of 30% sucrose buffer was carefully filled into the bottom of the tube using a needle-long syringe to keep the interface of the buffer as stable as possible. Sucrose buffer contains 2.38 g of HEPES, 10.17 g of MgCl_2_⋅6H_2_O, 200 g (20%) or 300 g (30%) of sucrose, and 0.15% DDM per liter of deionized water (pH 8.0). The pH was adjusted to 8.0 with sodium hydroxide solution. The tube was capped with parafilm and allowed to stand at room temperature for 2 h in a tilted state to form a sucrose density gradient. Thereafter, it was left for 1 h at 4°C. One milliliter of solubilized membrane protein (10 mg/ml) solubilized from the β-His strain was carefully overlaid on the sucrose density gradient. Ultracentrifugation was performed using OptimaTML-80XP and an SW40 Ti Rotor (Beckman coulter) at 40,000 rpm at 4°C for 16 h. After ultracentrifugation, 400 μl of the fraction was carefully separated from the upper layer of the tube, and *A*_280_ of each fraction was measured with NANO DROP 200c (Thermo Fisher Scientific). Fractions in which cytochrome oxidase activity was observed using TMPD were used for the next analysis.

### Immunoblot Analysis of the β Subunit of F_1_-ATPase and CtaC Subunit of *caa*_3_-Type Terminal Cytochrome Oxidase From Fractions Separated by Sucrose Density Gradient Ultracentrifugation

A 15% acrylamide gel was prepared, and a sample buffer was added to each of 23 fractions in which cytochrome oxidase activity was observed and electrophoresed at 30 mA. Blue Star Prestained Protein Marker (NIPPON Genetics) was used as a marker. Proteins in the gel were transferred to nitrocellulose filters (Bio-Rad) by applying electricity at 20 V for 16 h in Tris-glycine-methanol buffer [25 mM Tris, 192 mM glycine, and 20% (v/v) methanol (pH 8.3)] using a Mini Trans-Blot^®^ Cell manufactured (Bio-Rad). Western blots were performed as described previously (Morino et al., 2008). The β subunit-His_6_ protein of ATP synthase was detected by anti-His antibody HRP conjugate (Qiagen). For detection of the CtaC protein, rabbit anti-CtaC polyclonal antibody (Eurofins Genomics) was used as a primary antibody and goat anti-rabbit IgG-HRP conjugate (Abcam) was used as a secondary antibody. ECL solution (Promega) was the usual detection reagent. A quantitative imaging system, ChemiDoc^TM^ XRS^+^ (Bio-Rad) and a PC application software, Quantity One were used for the detection and analysis of chemiluminescence images.

### Pull Down Assay and Immunoblot Analysis

One milliliter of Ni-NTA resin (QIAGEN) was packed in the column. One column volume is 0.5 ml. Two to four column volumes of distilled water were passed through the resin and subsequently 10 column volumes of wash buffer (10 mM HEPES, 5 mM MgCl_2_, 20 mM imidazole, 20 mM NaCl and 0.15% DDM, pH 8.0) were applied to the column to wash the resin. Imidazole was added to the membrane protein of the β-His strain solubilized by DDM to a final concentration of 20 mM and this was applied to the resin. The resin and solubilized membrane protein were well mixed, transferred to a 15 ml tube, and shaken at 4°C for 1 h with a nutator at low speed. Mixed resin and membrane protein were passed through the column to obtain a non-adsorbed fraction. Subsequently, 1 ml of wash buffer was passed through the column to obtain a washed fraction. Finally, 1 ml of elution buffer (10 mM HEPES, 5 mM MgCl_2_, 200 mM imidazole, 20 mM NaCl and 0.15% DDM, pH 8.0) was passed through the column, and the eluted fraction was obtained.

The solubilized fraction, non-adsorbed fraction, washed fraction, and eluted fraction obtained by the pull-down assay were used for immunoblotting analysis which was performed in the same manner as described above.

## Results and Discussion

### Verification of the Interaction Between Cytochrome *caa*_3_-Type Terminal Oxidase and F_1_F_o_-ATP Synthase

Sucrose density gradient ultracentrifugation and pull-down assays were carried out to verify the direct interaction between F_1_F_o_-ATP synthase and cytochrome *caa*_3_ type terminal oxidase involved in OXPHOS. Indirect interaction between F_1_F_o_-ATP synthase and cytochrome *caa*_3_ type terminal oxidase of *B. pseudofirmus* OF4 has been demonstrated by using saturated mobile electron spin resonance and differential scanning calorimetry analysis (Liu et al., 2007). However, there is no direct report that the two proteins form a complex. Sucrose density gradient centrifugation and pull-down assay were conducted to confirm the interaction of proteins under mild conditions to verify whether these two complexes form a complex in the cell membrane.

From the results of sucrose density gradient ultracentrifugation and its immunoblot analysis, monomeric cytochrome *caa*_3_ type terminal oxidase was detected in low molecular weight fractions, and F_1_F_o_-ATP synthase and cytochrome *caa*_3_ type terminal oxidase were simultaneously detected in the high molecular weight fractions (**Supplementary Figure S1**). This result suggested the possibility of interaction between these two protein complexes. Subsequently, we attempted a pull-down assay to detect direct interactions between them (**Supplementary Figure S2**). However, the cytochrome *caa*_3_-type terminal oxidase was not purified together with the F_1_F_o_-ATP synthase. This result suggests that interaction between the two complexes is not strong in the cell membrane, i.e., may be a weak protein interaction. It is also possible that there may be another membrane protein that mediates between the two complexes.

### Bioinformatics Analysis of BpOF4_01690

The results of the BLAST sequence analysis^3^ showed that the homologous small proteins of BpOF4_01690 are present predominantly in alkaliphilic *Bacillus* species (**Table 2**). The secondary protein structure prediction and hydropathy profile of BpOF4_01690 and its homologs indicated that each protein has two transmembrane-spanning segments and there are highly conserved charged amino acid residues in the loop region between the transmembrane segments (**Figures 2B,C**). However, no functional motif and domain were identified, and its physiological function remains unknown.

**Table 2:** Result of protein BLAST analysis against BpOF4_01690.

Strain	Protein names	GenBank	Length	Identity
		accession no.		(%)
*Bacillus pseudofirmus* OF4	BpOF4_01690	ADC48406.1	59	100
*Bacillus marmarensis* DSM 21297	A33I_13875	ERN53048.1	59	100
*Bacillus halodurans* C-125	BH2819	BAB06538.1	62	66
*Bacillus selenitrieducens* ATCC 700615	Bsel_2302	ADH99805.1	60	69
*Bacillus akibai* JCM 9157	JCM9157_3465	GAE36304.1	62	69
*Bacillus hemicellulosilyticus* JCM 9152	JCM9152_3343	GAE31848.1	60	63
*Bacillus* sp. TS-2	BTS2_0672	GAF63780.1	63	67
*Bacillus cellulosilyticus* DSM 2522	Bcell_1668	ADU29931.1	60	67
*Bacillus alcalophilus* ATCC 27647	BALCAV_0219525	KGA95903.1	61	62
*Bacillus wakoensis* JCM 9140	JCM9140_4660	GAE28435.1	62	62

*As a result of the BLAST search analysis using Uniprot, it was found that 250 strains belonging to the Firmicutes have homologous proteins of BpOF4_01690. Ten strains with top 10 scores (values considering similarity and expectation values) were extracted in order. All 10 strains were alkaliphilic *Bacillus* spp.*

### Growth of the Wild Type, Δ01690, and Δ01690-R Under Low-Sodium Conditions

KGYE (potassium glucose-yeast extract) and KMYE (potassium malate-yeast extract) were used as growth media, in which K^+^ was used instead of Na^+^ at pH 7.5 and 10.5 (Wang et al., 2004). The major carbon sources in the KGYE and KMYE media were D-glucose and L-malic acid, respectively. In the KGYE medium, glucose was metabolized via the glycolytic pathway, and ATP was synthesized by OXPHOS and substrate-level phosphorylation. In contrast, in the KMYE medium, malic acid was metabolized via the TCA cycle, and ATP was synthesized predominantly by OXPHOS (**Supplementary Figure S3**).

**FIGURE 3:**
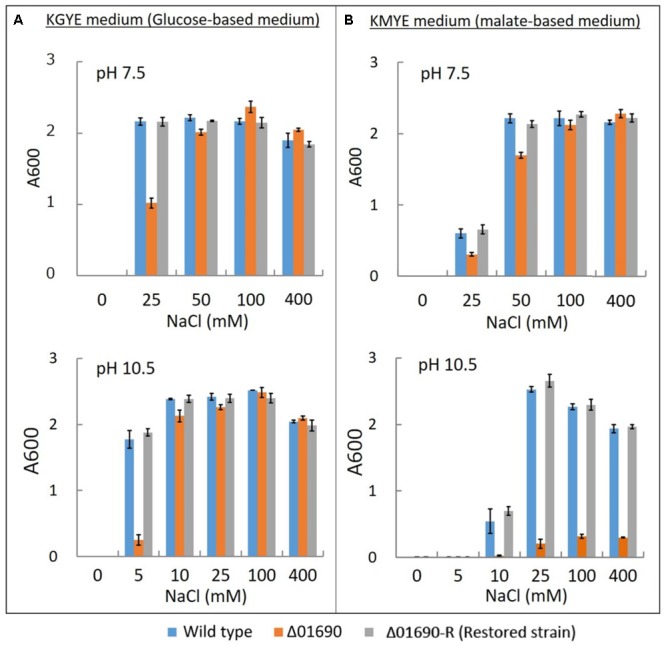
Growth of *B. pseudofirmus* OF4 (wild type), Δ01690, and Δ01690-R under various sodium concentrations. As preculture, each cell was grown in a GYE medium (pH 7.5) overnight at 37°C. Absorbance at A_600_ of each preculture was measured, and the A_600_ of each preculture was adjusted to 1.0. Next, each preculture was harvested by centrifugation and resuspended using the same medium as in the culture so that the glucose and Na^+^ were not transferred from the preculture to the culture. **(A,B)** Preculture (2 μl) was added to 2 ml of KGYE medium (pH 7.5), KGYE medium (pH 10.5), KMYE medium (pH 7.5), and KMYE medium (pH 10.5) with various concentrations of NaCl and grown aerobically at 37°C for 16 h. The A_600_ of the cultures was then measured. The error bars indicate standard deviations for the results from duplicate cultures in three independent experiments.

Alkaliphilic bacteria generally require Na^+^ for growth. Reportedly, Na^+^ in the medium is utilized as a source of coupling ions for flagellar rotation, uptake of various substrates, Na^+^/H^+^ antiporters, voltage-gated sodium channel, etc. (Krulwich and Ito, 2013; Preiss et al., 2015; Ito et al., 2017; Morino et al., 2017). Therefore, ensuring optimal Na^+^ concentration is critical for the provision of favorable growth conditions. Earlier reports showed that higher NaCl concentrations were required at pH 7.5 than at pH 10.5 to support optimal growth rates (Ito et al., 1997). Thus, to determine the effect of Na^+^, K^+^ was used as a substitute for Na^+^ in the KGYE and KMYE media. Then, growth experiments with various concentrations of added Na^+^ were conducted (**Figure 3**) (Terahara et al., 2012).

The growth of the wild type, Δ01690, and Δ01690-R in the KGYE medium at pH 7.5 was almost identical to the growth with the addition of 50 mM Na^+^. However, 50% of the growth of Δ01690 was observed at 25 mM Na^+^ compared with wild type and Δ01690-R (**Figure 3A**). Moderate decline in growth of Δ01690 in the KMYE medium at pH 7.5 was observed under 25 mM and 50 mM Na^+^ conditions compared to wild type and Δ01690-R (**Figure 3B**). In contrast, the growth of the wild type, Δ01690, and Δ01690-R in the KGYE medium at pH 10.5 was almost identical to that in the medium with the addition of 10 mM Na^+^. Nevertheless, at 5 mM Na^+^, the growth of Δ01690 was poorer than that in the wild type and Δ01690-R (**Figure 3A**). Poor growth of Δ01690 was observed under all tested conditions in the KMYE medium at pH 10.5. Both the wild type and Δ01690 grew well in NaCl concentrations over 25–400 mM (**Figure 3B**).

### Comparison of the Expression Level of Protein BpOF4_01690 Under Different Growth Conditions

The expression level of BpOF4_01690 fused with 6xHis-tag in the strain Δ01690-R-His_6_ cultured in KMYE and KGYE media at pH 10.5 was detected by western blotting (**Supplementary Figure S4**). The highest expression level was detected when the cells were grown on KMYE medium containing 25 mM Na^+^ at pH 10.5. However, no dramatic increase or decrease in the protein expression was detected under either condition.

### Measurements of Diverse Respiratory Chain Activities and Expression Levels of Cytochrome *bc*_1_ of the Wild Type, Δ01690, and Δ01690-R

**FIGURE 4:**
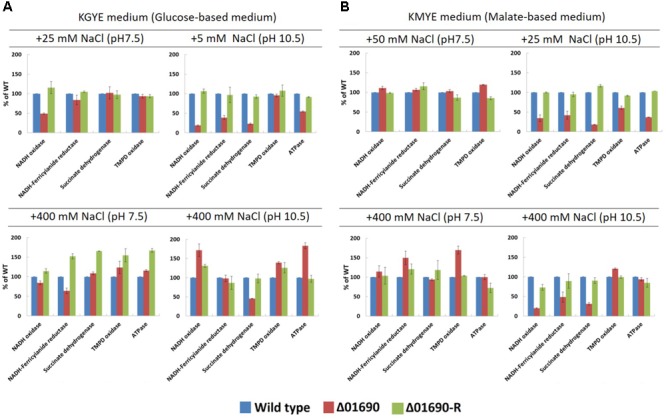
Measurements of various respiratory chain activates of *B. pseudofirmus* OF4 (wild type), Δ01690, and Δ01690-R under various growth conditions (**A**: KGYE medium, **B**: KMYE medium) at pH 7.5 and 10.5. Based on the specific activity of the wild type, referred to as 100%, the specific activities of Δ01690 and Δ01690-R (restored strain) are shown as relative activities (%). The error bars indicate standard deviations for the results from three independent experiments. The details of the experiment are described in the “Materials and Methods” section.

Under the condition that the growth of Δ01690 is worse than that of the wild type, enzymatic activities of various respiratory chain complexes of the wild type were measured in both Δ01690 and Δ01690-R under high- and low-sodium conditions at pH 7.5 and 10.5 (**Figure 4**). The activities of NADH oxidase, NADH ferricyanide reductase, succinate dehydrogenase, TMPD oxidase, and F_1_F_o_-ATPase were lower than those in the wild type in the KMYE medium plus 25 mM NaCl at pH 10.5 (**Figure 4B**, upper right). The high NaCl concentration (400 mM) in the KMYE medium at pH 10.5 enabled recovery of the activity of TMPD oxidase and ATPase (**Figure 4B**, bottom right). Both KGYE medium plus 5 mM NaCl at pH 10.5 and KMYE medium plus 25 mM NaCl at pH 10.5 showed similar phenotype except TMPD oxidase activity (**Figure 4A**, upper right and **Figure 4B**, upper right). The high NaCl concentration (400 mM) in the KGYE medium at pH 10.5, the activity of NADH oxidase, TMPD oxidase and ATPase exhibited increased up to 172% ± 17%, 139% ± 3% and 183% ± 8%, respectively, compared with wild type (**Figure 4A**, bottom right). On the other hand, the activity of succinate dehydrogenase was decreased to 45% ± 1% compared with wild type. These results suggest that at highly alkaline pH, the protein BpOF4_01690 affects both the respiratory chain and ATP synthesis by OXPHOS.

**FIGURE 5:**
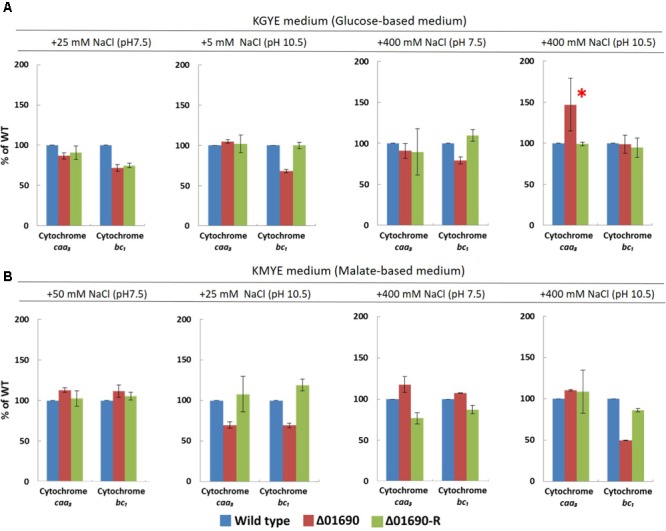
Expression levels of cytochrome *caa*_3_ and cytochrome *bc*_1_ of *B. pseudofirmus* OF4 (wild type), Δ01690, and Δ01690-R under various growth conditions (**A**: KGYE medium, **B**: KMYE medium) at pH 7.5 and 10.5. Based on the expression level of the wild type, referred to as 100%, the expression levels of Δ01690 and Δ01690-R (restored strain) are shown as relative activities (%). The error bars indicate standard deviations for the results from three independent experiments. The detailed description of the experiment is presented in the “Materials and Methods” section.

The expression levels of cytochrome *bc*_1_ and cytochrome *caa*_3_ of everted membrane vesicles prepared from the wild type, Δ01690, and Δ01690-R in the KGYE and KMYE media with low or high Na^+^ concentrations at pH 10.5 were determined by heme staining and compared (**Figure 5**). In the KGYE medium with 5 mM Na^+^ and pH 10.5, the expression level of cytochrome *bc*_1_ of Δ01690 was reduced to 68% ± 2% of that of the wild type (**Figure 5A**). In contrast, in the KGYE medium with 400 mM Na^+^ and pH 10.5, the expression level of cytochrome *caa*_3_ of Δ01690 increased to 147% ± 32% of that of the wild type (**Figure 5A**). However, under an identical condition, there was no indication that the growth of Δ01690 was more intensive than that of the wild type (**Figure 3A**, bottom). In the KMYE medium with 25 mM Na^+^ and pH 10.5, the expression levels of cytochrome *caa*_3_ and cytochrome *bc*_1_ of Δ01690 were reduced up to 70% ± 4% and 69% ± 3% of those of the wild type, respectively (**Figure 5B**). In contrast, in the KMYE medium with 400 mM Na^+^ and pH 10.5, the expression level of cytochrome *bc*_1_ of Δ01690 decreased to 49% ± 0% of that of the wild type (**Figure 5B**). These findings suggest that the deletion of BpOF4_01690 negatively affects the expression level of cytochrome *caa*_3_ in the KMYE medium with a low Na^+^ concentration and high pH; the expression level of cytochrome *bc*_1_ under all tested conditions was also influenced at high pH, except in the KGYE medium with 400 mM Na^+^ at pH 10.5.

### Growth of Strains Δ*ctaD* and ΔF_o_ Mutants Under Low-Sodium Conditions

**FIGURE 6:**
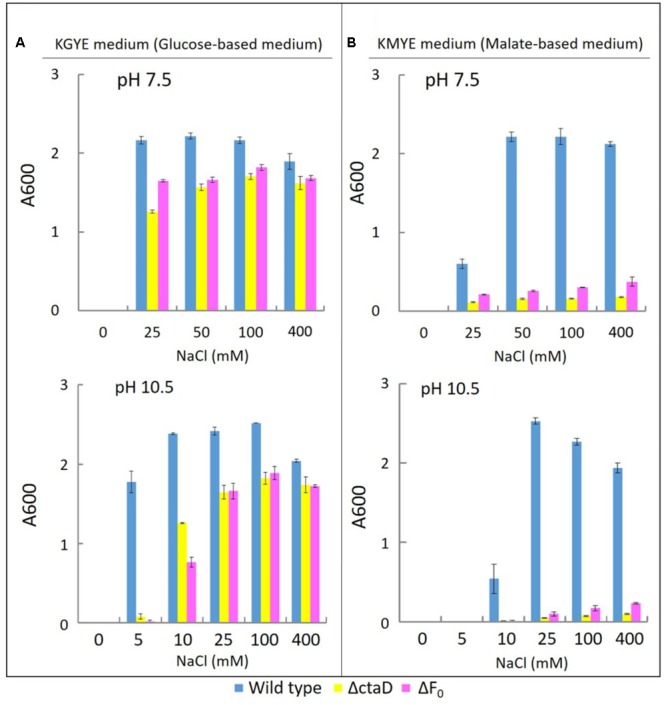
Growth of *B. pseudofirmus* OF4 (wild type), Δ*ctaD*, and ΔF_o_ under various Na^+^ concentrations (**A**: KGYE medium, **B**: KMYE medium). The experimental method used is the same as that outlined in the legend of **Figure 3**.

To compare the phenotype of Δ01690 with other respiratory chain and OXPHOS-related mutants, the Δ*ctaD* mutant in which disruption was caused in the *ctaD* of *caa*_3_-type terminal oxidase operon and the ΔF_o_ mutant with the deleted F_o_ part (*atpB-F*) of *unc* operon were used as reference and comparative strains. The growth of the wild type and these two mutants was measured in the KGYE and KMYE media at pH 7.5 and 10.5 (**Figure 6**).

The growth of the wild type, Δ*ctaD*, and ΔF_o_ in the KGYE medium at pH 7.5 and 10.5, was compared as a function of NaCl concentration (**Figure 6A**). The wild type had optimal growth at 25–400 mM NaCl at pH 7.5 and at 5–400 mM NaCl at pH 10.5, whereas both Δ*ctaD* and ΔF_o_ mutants had a significantly lower level of growth at 10 mM NaCl at pH 10.5 (**Figure 6A**). The poor growth of both Δ*ctaD* and ΔF_o_ mutants was observed in the KGYE medium with 5 mM NaCl and pH 10.5 and compared to that of the wild type (**Figure 6A**). Meanwhile, the growth of both Δ*ctaD* and ΔF_o_ mutants in the KMYE medium at both pH values was poor under all examined conditions, even at concentrations above 25 mM Na^+^, in which the wild type grew actively (**Figure 6B**).

### Measurements of Various Respiratory Chain Activities and Expression Levels of Cytochrome *bc*_1_ of Δ*ctaD* and ΔF_o_ Mutants

**FIGURE 7:**
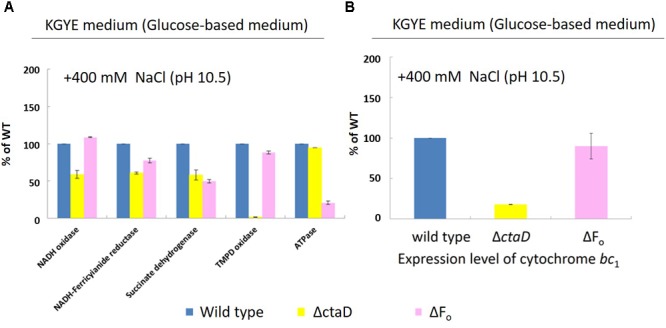
Measurements of various respiratory chain activities **(A)** and the expression level of cytochrome bc1 **(B)** of *B. pseudofirmus* OF4 (wild type), Δ*ctaD*, and ΔF_o_ in the KGYE medium plus 400 mM Na^+^ at pH 10.5. Based on the specific activity of the wild type, referred to as 100%, the specific activities of Δ*ctaD* and ΔF_o_ are displayed as relative activities (%). The error bars indicate standard deviations for the results from three independent experiments. The details of the experiment are described in the “Materials and Methods” section.

Measurements were performed of the activities of various respiratory chain complexes and the expression levels of cytochrome *bc*_1_ of the wild type, Δ*ctaD*, and ΔF_o_ in the KGYE medium with 400 mM Na^+^ and pH 10.5 (**Figure 7A**), followed by comparative assessments. The enzymatic activity of NADH oxidase, NADH-ferricyanide reductase, succinate dehydrogenase, and TMPD oxidase, as well as the expression level of cytochrome *bc*_1_ in Δ*ctaD*, were lower than those of the wild type. In particular, the activity of TMPD oxidase was hardly detected. In contrast, the ATP hydrolysis activity of Δ*ctaD* was almost identical to that of the wild type.

For strain ΔF_o_, the enzymatic activities of NADH-ferricyanide reductase, succinate dehydrogenase, and ATPase were lower than those of the wild type. In particular, the ATPase activity was drastically reduced. In contrast, the enzymatic activity of NADH oxidase and TMPD oxidase, as well as the expression level of cytochrome *bc*_1_, were almost identical to those of the wild type.

The expression level of cytochrome *bc*_1_ of Δ*ctaD* and ΔF_o_ mutants, one of the terminal oxidases indicated that the Δ*ctaD* mutant was much lower in activity compared with the wild type and the ΔF_o_, which showed little activity loss (**Figure 7B**).

**FIGURE 8:**
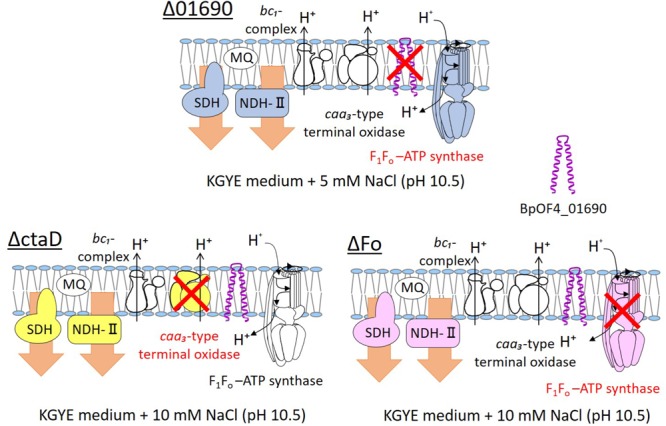
Thematic diagram of the phenotype summaries of the respiratory chain complexes of Δ01690, Δ*ctaD*, and ΔF_o_. Based on the results depicted in **Figures 4A, 7A**, the respiratory chain enzymes and ATP synthase of Δ01690, ΔctaD, and ΔF_o_ in the KGYE medium plus 5 mM NaCl (pH 10.5) are shown. The ΔctaD strain is a defective mutant of *caa*_3_-type terminal oxidase. The ΔF_o_ strain is a defective mutant of F_1_F_o_-ATP synthase. A common phenotype of all three strains was the decreased activity of SDH and NDH-II. BpOF4_01690 is shown as a purple structure.

We presumed that Δ*ctaD* influences the activities of multiple enzymes of the respiratory electron transport system. In contrast, ΔF_o_ displayed poor ATPase activity and reduced levels of both NADH dehydrogenase and succinate dehydrogenase. The levels of Δ*ctaD* and ΔF_o_ were indirectly influenced by the actions of multiple enzymes of the respiratory electron transport system. A thematic diagram of the phenotypes of the respiratory chain complexes of Δ01690, Δ*ctaD*, and ΔF_o_ at high pH is illustrated in **Figure 8**. Importantly, a deletion of either Δ*ctaD* or ΔF_o_ reduced the expression of the electron transfer enzymes, SDH and NDH-II. Moreover, the deletion of *ctaD* also led to the loss of *caa*_3_-type terminal oxidase activity.

In view of the observations recorded above, similarly to Δ*ctaD* and ΔF_o_ mutants, the negative effect of the enzymatic activity of respiratory chain complexes of Δ01690 might have been due to independently exerted effects that directly influenced the *caa*_3_-type terminal oxidase or F_1_F_o_-ATP synthase (**Figure 8**). Therefore, we suggest that the deletion of BpOF4_01690 influences the activity of the respiratory chain-related enzymes and ATP synthesis by OXPHOS. Moreover, the small protein BpOF4_01690 may also play a critical role under lower sodium motive force conditions.

**FIGURE 9:**
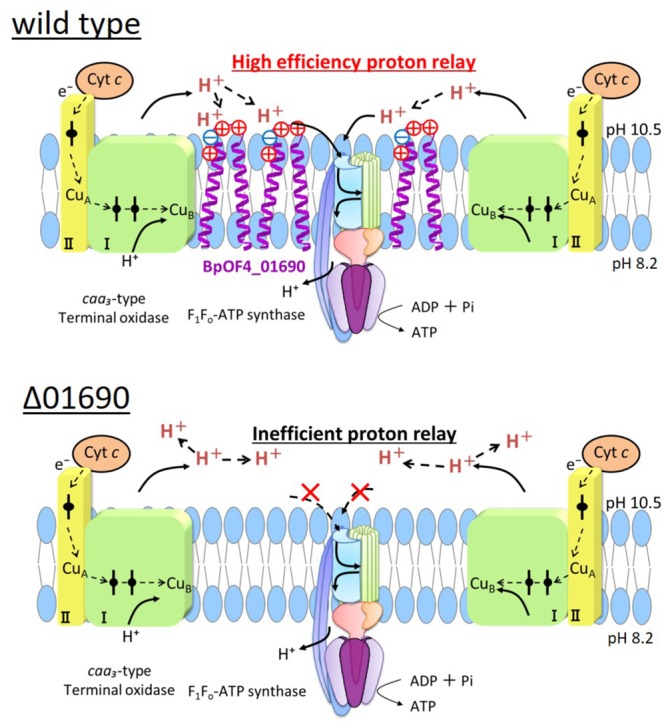
Model diagram of ATP synthesis by OXPHOS in the wild type and Δ01690 under highly alkaline conditions. In the wild type strain, OXIPHOS is conducted by efficient proton delivery between the respiratory chain and F_1_F_o_-ATPase due to the presence of BpOF4_01690. In contrast, OXIPHOS does not work well in the Δ01690 strain because protons from the respiratory chain are not transferred efficiently to F_1_F_o_-ATPase. Due to this negative influence, the growth in the KMYE medium was extremely poor. It is, therefore, inferred that BpOF4_01690 mediates this efficient proton delivery.

Highly conserved charged amino acid residues are present in the loop region between the transmembrane segments of BpOF4_01690 and its homologous proteins. Thus, we hypothesize that the negatively charged amino acid residue BpOF4_01690-E21 has a functionally critical role in the surrounding conserved positively charged amino acid residues (**Figure 2B**). We propose a working model describing the function of BpOF4_01690 (**Figure 9**). Some of the protons effluxed from the proton pump of the respiratory chain bind to negatively charged sites of the side chain of the glutamic acid residue of BpOF4_01690 at the outer surface of the cell membrane. Then, the protons are efficiently transferred to F_1_F_o_-ATP synthase, which is present in the proton pump of the terminal oxidase and BpOF4_01690 in the vicinity of a highly alkaline environment.

## Conclusion

In conclusion, the small protein BpOF4_01690 appears to play a central role in the energy-coupled retention of protons needed for ATP synthesis via OXPHOS of alkaliphilic *Bacillus* species in highly alkaline environments. This finding is very interesting while considering that alkaliphiles acquired BpOF4_01690 in the process of evolution to adapt to OXPHOS in alkaline environment.

## Author Contributions

TK and MI designed the research. TT performed the research with experimental work. TT, TK, and MI analyzed the data. TK and MI wrote the paper.

## Conflict of Interest Statement

The authors declare that the research was conducted in the absence of any commercial or financial relationships that could be construed as a potential conflict of interest.

## Footnotes

1 https://blast.ncbi.nlm.nih.gov/Blast.cgi?PROGRAM=blastp&PAGE_TYPE=BlastSearch&LINK_LOC=blasthome

2 https://www.genome.jp/tools-bin/clustalw

3 http://blast.ncbi.nlm.nih.gov/Blast.cgi
