# Supplementary material for: A Hydrophobic Small Protein, BpOF4_01690, Is Critical for Alkaliphily of Alkaliphilic Bacillus pseudofirmus OF4
Source: Front Microbiol. 2018 Aug 28;9:1994. doi: 10.3389/fmicb.2018.01994 (PMC6120979; doi:10.3389/fmicb.2018.01994)
Supplement: Supplementary file 1 [file Data_Sheet_1.PDF]

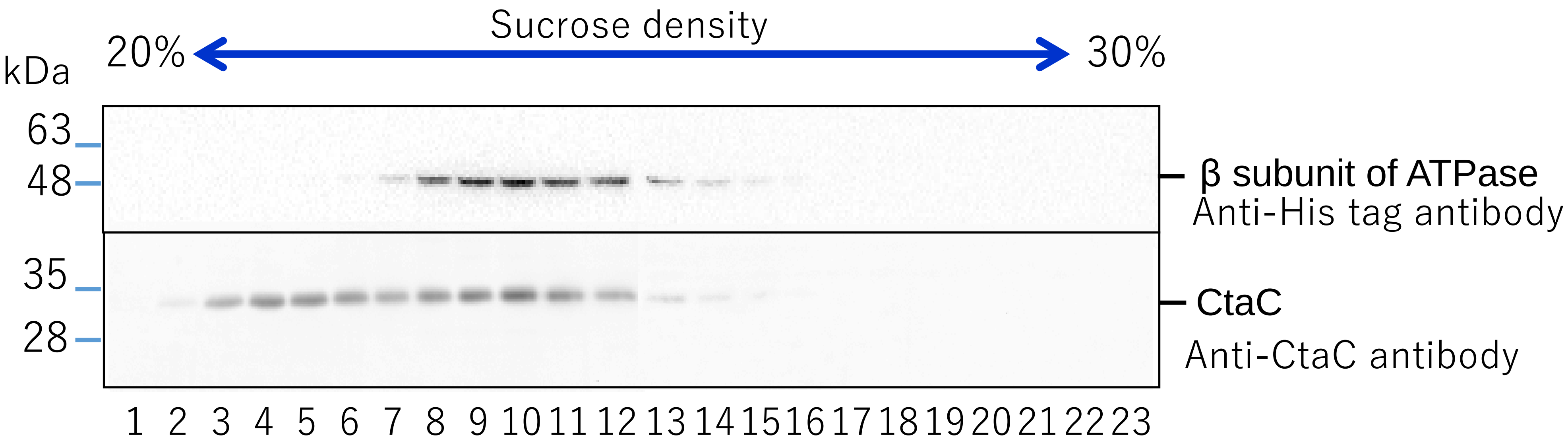


**Supplemental Figure S1. Detection of F1Fo-ATP Synthase and Cytochrome *caa*$_3$ Type Oxidase by Sucrose Density Ultracentrifugation Using Solubilized Membrane Protein of β-His Strain of *B. pseudofirmus* OF4.**

The ATP synthase with the 6xHis tag added to the β subunit was purified on a Ni-NTA column. Anti-histidine tag antibody recognizing β subunit of ATP synthase and Anti-CtaC antibody recognizing subunit II (CtaC) of cytochrome *caa*$_3$ type oxidase, respectively. The details of the experiment are described in the materials and methods.

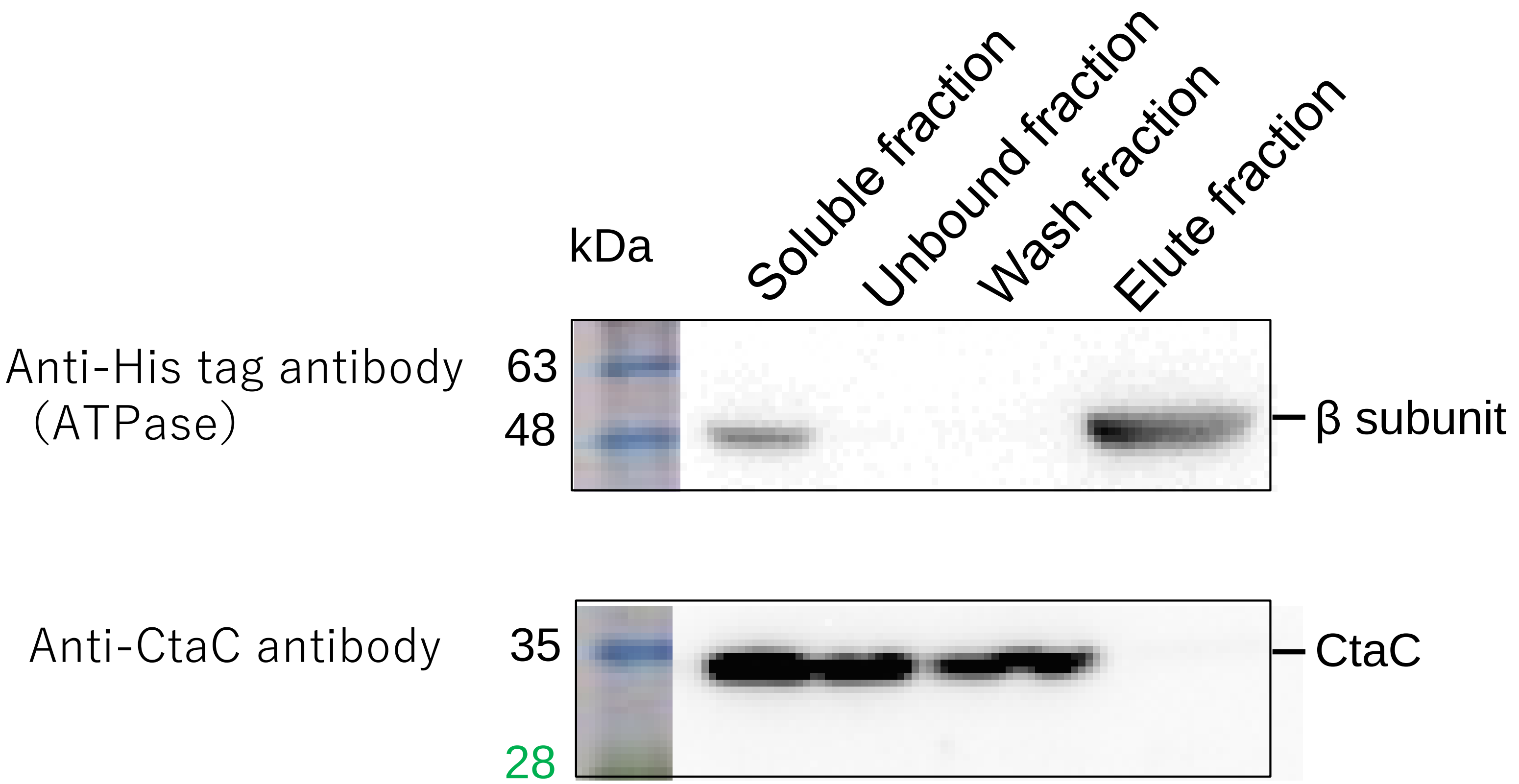


**Supplemental Figure S2. Verification of the Interaction Between $F_1F_O$-ATP Synthase and Cytochrome *caa*$_3$ Type Oxidase by Pull-Down Assay**

The ATP synthase with the 6xHis tag added to the β subunit was purified on a Ni-NTA column. Anti-histidine tag antibody recognizing β subunit of ATP synthase and Anti-CtaC antibody recognizing subunit II (CtaC) of cytochrome *caa*$_3$ type oxidase, respectively.

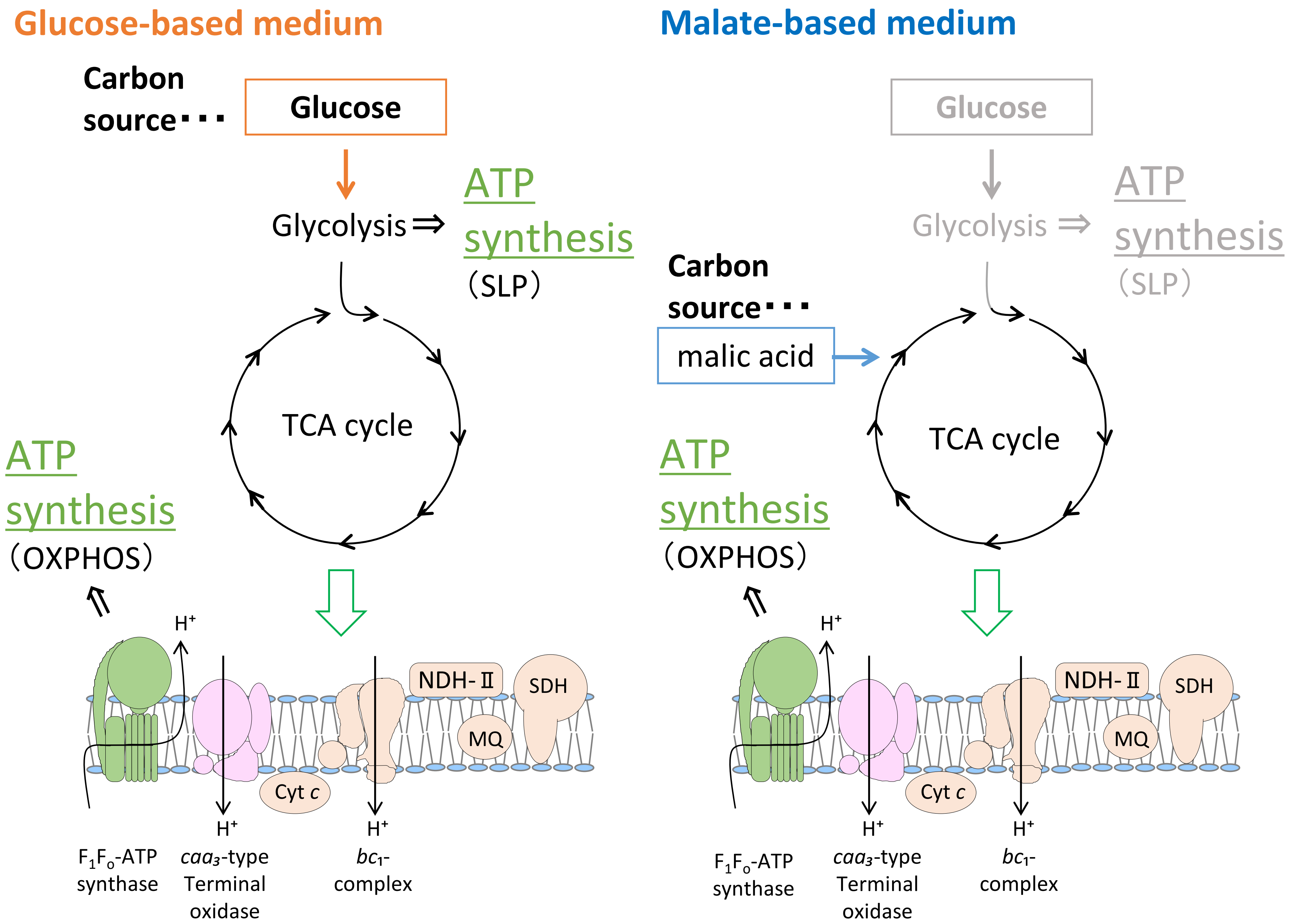


**Supplemental Figure S3. Effect of the Carbon Source Used in Glucose- and Malate-based Media on ATP Synthesis in the Alkaliphilic *Bacillus pseudofirmus* OF4.** Abbreviations: SLP: substrate-level phosphorylation, OXPHOS: oxidative phosphorylation.

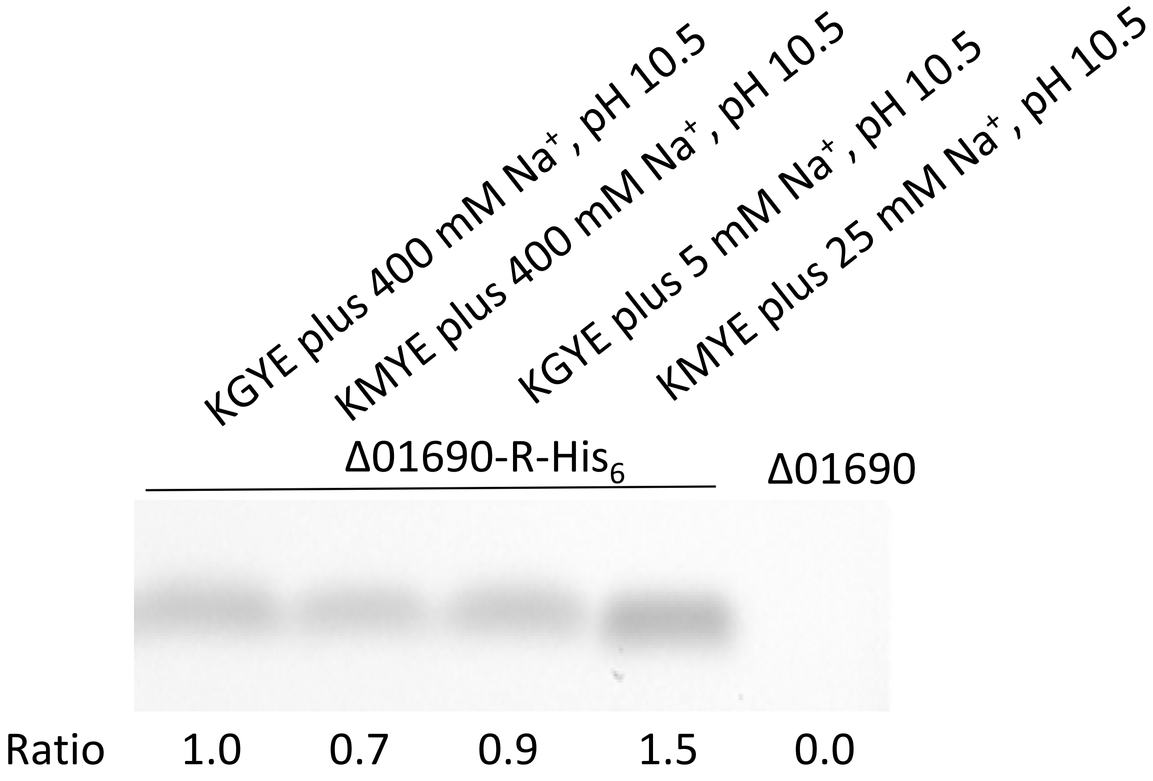


**Supplemental Figure S4. Detection of the Expression Level of BpOF4_01690 Using the 6xHis-tag of Strain Δ01690-R-His$_6$ Under Diverse Growth Conditions.**

The detailed description of the experiment is provided in the materials and methods. The ratio of the expression level of BpOF4_01690 under various conditions is described when the expression level of KGYE plus 400 mM $Na^+$ at pH 10.5 is set to 1.
